# The role of grid management in community risk governance: a case study in Yuelu, China

**DOI:** 10.3389/fpubh.2024.1353890

**Published:** 2024-05-16

**Authors:** Shan Gao, Wenhui Liu

**Affiliations:** ^1^School of Public Administration, Central South University, Changsha, China; ^2^School of Economics, Management and Law, University of South China, Hengyang, China

**Keywords:** grid management, social risk, community risk governance, social safety valve, community public health

## Abstract

**Objective:**

In this study, we aim to provide a comprehensive analysis of the effectiveness of the risk prevention and control mechanism within the grid management model for community risk prevention. We emphasize the importance of thoroughly examining the risk prevention and control mechanism to enhance risk management efforts in urban communities, particularly in response to unforeseen outbreaks such as COVID-19.

**Methods:**

Case studies are widely acknowledged as one of the most effective approaches to examine governance in China. In this study, the “Yuelu Model” serves as an illustrative example to demonstrate the application and effectiveness of grid management in community risk governance. To ensure the validity of the case study, it is imperative to adhere to the principle of representativeness. The collection of case data involves a combination of primary and secondary sources, and supplementary information is obtained through follow-up investigations conducted via WeChat, telephone, and other means, thereby enhancing the comprehensiveness and accuracy of the data.

**Results:**

Our analysis reveals significant findings regarding the impact of the grid management model, fulfilling a triple role as a “Social Safety Valve” in the management process: (1) Community stress reduction function, (2) Community alarm function, and (3) Community integration function. Furthermore, we explore the adaptability of the grid management mechanism in addressing community risks, highlighting its effectiveness and potential for broader application.

**Discussion:**

The findings of this study suggest that: Firstly, it is crucial to establish a shared information repository among different departments on a big data platform. Secondly, a dynamic government public information internal network should be established through collaborative efforts among multiple departments. Thirdly, implementing a regular (or periodic) early warning mechanism is essential. Lastly, the establishment of a high-quality talent team for power grid management is highly recommended. Our research provides valuable insights to enhance community risk governance.

## 1 Introduction

### 1.1 Background and objective

The outbreak and rapid spread of the Corona Virus Disease 2019 (COVID-19) continually challenge the management capacity and pandemic prevention measures of urban communities in China. As China’s economic and social system reform progresses, certain long-standing social conflicts are gradually surfacing and escalating. The Third Plenary Session of the 18th Communist Party China Central Committee proposed innovative reforms to the social governance system. The proposed direction includes focusing on grid management and social services, as well as improving the comprehensive service management platform at the grassroots level. In essence, grid management can be seen as a responsive approach to address social management challenges arising from ongoing social changes. The societal structure prior to 1978 underwent significant transformation due to the influence of modernization forces, including market economic relations and political system reforms. The unit-based management approach faced challenges in adapting to the demands of market economic development. Additionally, the high population density and mobility within cities further compound these challenges, the mobility of the population, the high degree of differentiation of urban social groups, these factors pose significant challenges to urban social management (1). Therefore, grid management has emerged under the situation of frequent social conflicts and the proliferation of social contradictions.

The sudden and irregular nature of major public health events necessitates a response that combines flexibility and regularity. In the case of a sudden outbreak of a pandemic, focused control becomes imperative. There are three primary sets of contradictions to consider. Firstly, the randomness of disasters contradicts the regularity of traditional governance; second, the suddenness of major public health events contradicts the flexibility of government governance; and third, the comprehensiveness of outbreaks contradicts the lack of public services. The traditional top-down management approach is insufficient to meet real needs. Hence, the introduction of grid management becomes imperative. Grid management exhibits distinct characteristics, including horizontal expansion to the edges, vertical integration to grassroots levels, and comprehensive coverage across various domains.

Throughout the stages of human society, risk has been an inherent element. However, with the expansion of human activities and increased interactions, the impact of human actions and decisions on nature and society itself has grown, leading to heightened uncertainty and risk. Renowned sociologist Niklas Luhmann aptly described our current reality as a “society where there is no alternative to risk-taking” (2). Ulrich Beck further emphasized that, following the decline of traditional societies, humanity finds itself in a “risk society” during the process of modernization and globalization (3). Conceptually, risk is deeply embedded in modern society and is intertwined with human choices, behaviors, and various technical and institutional factors. The application of science and technology, social culture, legal systems, and other elements create uncertainties, potential dangers, and harms within social behavior and order. In a highly modernized societies, risk transcends individual behavior and extends beyond geographical and socio-cultural boundaries. The rapid development of advanced technology, expanding globalization, the strain on natural resources, and cultural clashes have introduced significant uncertainties and high risks to the domains of politics, economy, and culture in modern society.

Risk has become a pervasive phenomenon, present in all aspects of social development. Risk is not only shaped by the natural, political, and institutional environments in which individuals reside but also arises from collective and individual decisions, choices, and actions (4). Moreover, scholars have noted that social risks are closely intertwined with economic activities. As economic interactions become more frequent and economic development accelerates, conflicts of interest are more likely to arise. These conflicts increase the costs associated with social interactions, alter the overall sense of security and trust in society, and ultimately lead to heightened social tensions (5). Under the grid management model, the interconnection between the participating subjects and the expansion of social capital’s influence contribute to the heightened complexity of social risks. Risk has become an defining characteristic of modern society.

Therefore, the attention given to risk theory and related issues can be perceived as a reflection and conscious response of human society towards the matters of self-existence and development (6). The significance of risk theory lies in its provision of a new theoretical perspective that aids in comprehending the evolution, prevention, and control of risks across various domains within modern society. China is presently undergoing a phase of social transformation, and various conflicts of interest and conflicts and disputes are showing a proliferation. By bolstering the study of risk theory, raising risk awareness and crisis consciousness among societal actors, and enhancing the overall risk management and prevention capabilities of the entire society, the public can be equipped with scientific means to preempt and address diverse unexpected risks. This, in turn, enables a more rational response to social risks, adjustment of behaviors, and assurance of harmonious and sustainable societal development.

The grid management model is gaining prominence, and the majority of scholars extol its electronic and informatization features, as well as its capacity for speed and efficient service delivery through organizational innovation. However, there has been limited investigation into its mechanisms for risk prevention and control. Therefore, this paper focuses on comprehending risk governance in the grid urban community governance model. It seeks to determine the feasible and effective in risk prevention, and whether there are other risk points through a comprehensive analysis of its pathways.

### 1.2 Previous studies

As the term suggests, “grid” entails the partitioning of urban regions into administrative units known as “grids,” which function as the foundation for government management of local communities. In the early stages of grid management implementation, the key objective was to empower residents by fostering their self-management abilities and facilitating self-service initiatives. Additionally, it aimed to activate the latent potential for self-governance within society. Grid management, therefore, exemplifies the government’s aspiration to establish social order and foster harmonious coexistence within the community.

In academic research on grid risk governance, three main perspectives can be identified:

One is the analysis of risk evolution and stages in grid governance. By studying the process of risk evolution in complex projects and urban communities, different stages can be identified and the key elements and influencing mechanisms of each stage can be analyzed in depth (7). This evolutionary perspective reveals the process of risk governance from accumulation to amplification and then to crisis eruption, providing theoretical support for addressing challenges at different stages. Through the analysis of risk evolution in governance, targeted grid governance models can be developed to effectively address the accumulation and eruption of governance risks (8).

The second is the study of grid structure and collaborative governance. This perspective focuses on the operation and distribution of power in grid management, particularly the interaction between vertical and horizontal power (9, 10). Researchers can explore the impact of power structure on grid governance and the balance between vertical and horizontal power (11). Within this perspective, researchers can emphasize the importance of multi-stakeholder participation under government leadership through the study of collaborative governance models of power structure, aiming to achieve a balance in power structure and enhance the public nature of community governance (12).

The third is the study of technology and institutional dynamics in grid management. This perspective focuses on the role and impact of technology in grid governance and examines the influence of technological innovation on institutions (13). Researchers can investigate the effects of technology introduction in grid management and the institutional rigidity resulting from technology (14). To exemplify, community-based grid management plays an important role in identifying, screening, and referring infected persons with infectious diseases (15). They can explore ways to enhance institutional flexibility to adapt to the challenges brought by technological innovation (16–18). Specifically, this perspective highlights the establishment of flexible institutional rules, the encouragement of innovative practices, and the moderate introduction of technological support to promote the flexibility and innovative capacity of grid management.

In fact, the grid management approach has been widely implemented globally and has shown positive results in various cities and countries. For example, many cities in China have adopted grid management methods, including Shanghai, Guangzhou, Shenzhen, and others. These cities divide their urban areas into grid units to achieve more efficient urban management and service delivery. Through grid management, city administrators can have a more precise understanding of the needs and issues of the city and take targeted measures accordingly.

However, the existing literature exhibits a fragmented research trend, lacking a comprehensive explanation for the mechanism of grid management’s role in community risk prevention and control. Specifically, it fails to consider the integrated impact of risk structure and risk perception on risk transformation. In light of Lewis Coser’s conflict theory, this paper aims to address this gap by focusing on the application of grid management in community risk management. It seeks to explore how grid management facilitates effective risk prevention and control within communities.

## 2 Theoretical framework

This paper analyzes the risk governance of urban community grid management based on Lewis Coser’s social safety valve theory. The social conflict school posits that society is a complex system composed of interconnected components, giving rise to imbalances, tensions, and conflicts (19) among the connected parts. This conflict is a struggle over values, rare status, rights, and resources. There are both positive and negative functions of conflict, but unlike functionalism, which emphasizes the negative functions of conflict, social conflict theory focuses more on the positive functions of conflict and considers conflict as a norm. It is the social safety valve theory that expresses the positive functional point of conflict. In his safety valve theory, Lewis Coser states that “safety valves” are customs and institutions that provide institutionalized outlets for hostility and general internal drives that are repressed by the group, a socially sanctioned framework for conducting conflict without leading to the disruption of in-group relations, and a role as a channel for venting and releasing. Failure to provide such outlets for the release of hostility can damage the relationship between the opposing parties (20). Generally, social systems provide people with institutions to vent hostile and offensive emotions, i.e., safety valve systems, which help maintain the relationship between the opposing parties and preserve the stability of the system. The more rigid a social structure is, the more the value of the safety valve mechanism can be demonstrated. A rigid society is fragile and does not allow for conflict. If the abolition of the “safety valve” mechanism, hostility has nowhere to vent, then there will be a great danger to society once the outbreak will certainly cause structural damage to society. At present, China’s development is at an important strategic opportunity stage, with dramatic social changes, more conflicts within groups, many contradictions overlapping, and increased risks and hidden dangers. If we do not provide an effective channel for venting, it may cause a major crisis in the entire social structure. The grid community management model that we are going to explore in this paper plays the role of a safety valve in the current Chinese society, providing a means and a channel to vent the discontent in the social transformation, to remove the potential threat to the social structure, i.e., social risk. Gird governance aims to establish a novel and cohesivesocial management system through system integration, information integration, process re-engineering, performance evaluation, cooperative governance, and integrity management (21). This model is “a process re-engineering of public services, which provides a public service that is demand-driven, refined, personalized and comprehensive, and achieves a breakthrough and transcendence of seamless government” (12).

Lewis Coser’s social safety valve theory has the following three distinctive features: (1) allowing the existence of conflict. He argues that “conflict cleanses the air” and that a system that allows hostile feelings to escape while leaving mutual relations intact acts as a lightning rod. It prevents the accumulation of blocked hostile tendencies by allowing the free expression of behavior. (2) Social conflict points to alternative targets. Lewis Coser notes that “we use the term ‘safety valve’ to denote a system that directs discontent to alternative targets (or that provides the means for such diversion), rather than a system that allows conflict to manifest itself” (20). (3) Social safety valves are diverse. For example, trade associations, people’s congress systems, judicial mediation, social organizations, etc., and even political jokes can serve as safety valves, all of which can provide decompression energy for hostility.

The problem of risk in community management arises first from the risk of gaming between community parties. This type of risk is endogenous and man-made. The complex game among community residents or owners’ associations, owners’ committees, community neighborhood committees, community party branches, property management companies, community sanitation, and other service organizations increases the possibility of unspecified risks (22). The main risks are the tension between community autonomy and government domination, the lack of position, overstepping and misalignment caused by the interlocking functions between community committees and party branches, and the imbalance between community residents and the supply and demand of public goods, including material public goods and spiritual public goods, involving the supply and demand of necessities such as water, gas, electricity, network communication, and other service products. The risk of conflict between the owners of the community and the owners’ committee and the risk of conflict between the owners of the community and the property service companies is mainly caused by the dissatisfaction of the owners with the quality of property management and the service fees charged. The public revenue of the community is also of special concern to the owners, such as the public facilities and equipment, social organizations, etc.

**Figure 1:**
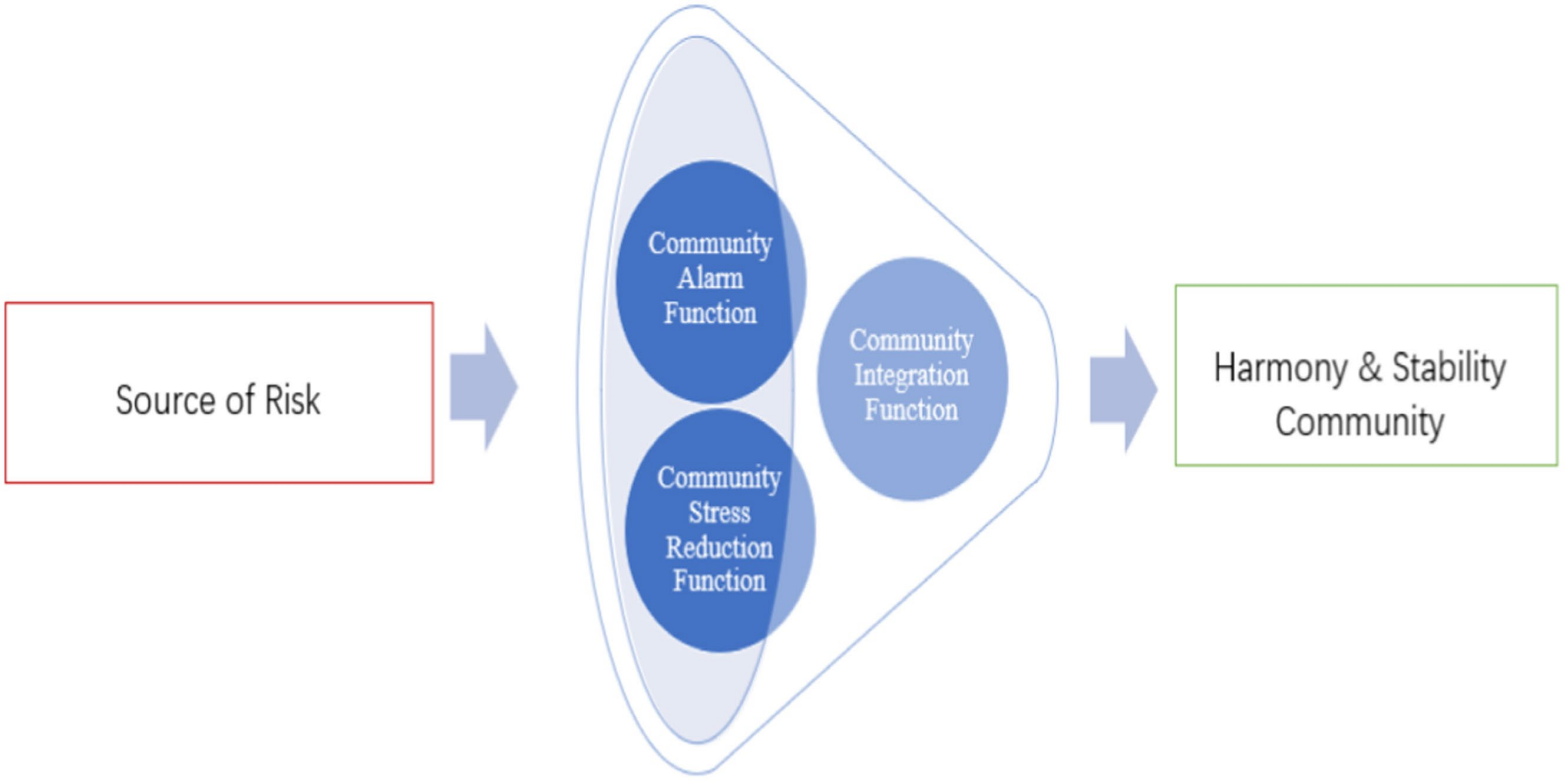
The role of grid management in community risk governance.

Institutional risks in community governance arise from the process of institutionalization and standardization. This includes hidden risks within the system, where experts involved in system creation may be aware of defects but downplay them when explaining the system to the public. Operational blockages and malfunctions can also generate related risks. The complexity of community building during the transition period leads to variability and instability, resulting in the absence, misalignment, and mismatch of systems. This not only increases system risks but also raises concerns about the credibility of the system itself, leading to moral risks among society members (23, 24). In addition, community management styles, tools, and mechanisms can also generate corresponding risks. Based on this, this paper discusses the “Social Safety Valve” function of grid management for communities from three perspectives: community stress reduction function, community alarm function, and community integration function, according to the expression of social conflict function (Figure 1).

## 3 Methodology and case introduction

### 3.1 Methodology

The case study approach facilitates the understanding of various aspects of the subject of the study, which leads to a comprehensive and in-depth understanding of the subject. Secondly, case studies help to clarify concepts and identify variables, thus facilitating further empirical research. It also helps to conduct exploratory research to identify important variables and provide useful categories to formulate hypotheses or build theories. Due to the extensive and in-depth information. Case studies facilitate an objective, in-depth, and accurate grasp of the problems and needs of the research subjects and their causal mechanisms. It is conducive to proposing effective and specific approaches or solutions to the problem.

This paper mainly applies a case study as a tool and selects the “Yuelu model” as the research object. Yuelu District has been awarded the title of “Top Ten Social Management Innovation,” ranking first in the country. Moreover, in the second national harmonious community building demonstration unit creation activities, the district Xianjiahu Street, Guanshaling Street, and five communities were awarded the national harmonious community building demonstration unit, and Yuelu District was evaluated as a national harmonious community building demonstration city; four units, including Changhua Community, were evaluated as national civilized units and civilized villages and towns.^1^ Thus, the study of the risk prevention and control mechanism of grid management in Yuelu District is representative. To find out the answers to the questions raised in this paper, the authors obtained survey information through many official documents, information retrieval, and collection.

### 3.2 Case introduction

**Figure 2:**
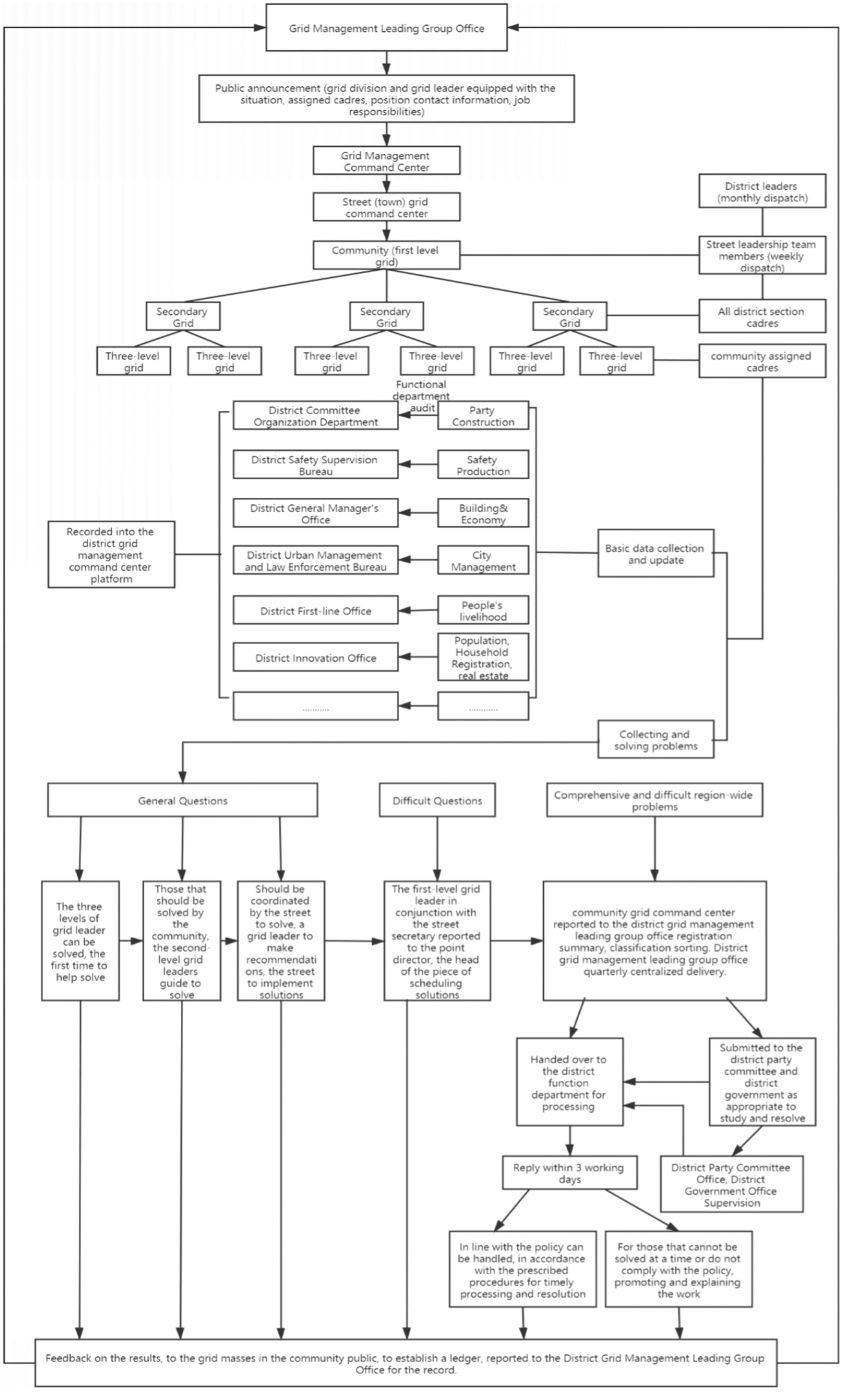
Flowchart of grid management work in Yuelu District.

Yuelu District is located on the west bank of the Xiangjiang River in Changsha, with 17 streets (towns), 167 communities (villages), a total area of 552 square kilometers, and a resident population of 820,000. Grid management is first established in the district’s 17 streets (towns) under the jurisdiction of communities (villages), scenic spots (parks), building a grid responsibility system based on communities (villages), scenic spots (parks) as the basic unit, the implementation of grid management, to maintain the normal long-term effect of urban management, to achieve the work content in the grid, responsibility in the grid, supervision in the grid, assessment in the grid work objectives. In recent years, Yuelu District has made useful explorations in social governance by adhering to source management, taking grid management and social services as the direction, taking “grid+” as the breakthrough, improving the comprehensive service management platform at the grassroots level, reflecting, and coordinating people’s interests and demands in various aspects and levels promptly, and innovating and continuously improving. Certain results have been achieved. The specific workflow is shown in Figure 2 below:

To maximize community mobilization, implement grid management, comprehensive prevention and control measures, and group prevention and control. This ensures early detection, reporting, isolation, diagnosis, and treatment, preventing the import, spread, and export of the pandemic while controlling its transmission. The community carries out appropriate prevention and control strategies and measures according to different pandemic situations (Table 1).

**Table 1:** Prevention and control strategies and measures for different pandemic situations in the community.

Pandemic Situation	Prevention and Control Strategy	Prevention and Control Measures
No cases found in the community	External anti-input	1. Organizational mobilization. 2. Health education. 3. Information informing. 4. Management of returnees from infected areas. 5. Environmental health management. 6. Material preparation.
Case or outbreak in the community spread of outbreak	Internal anti-diffusion, external anti-output Internal anti-spread, external anti-output	Above1-6. 7. Close contact management. 8. Disinfection. Above1-8. 9. Blockade of the infected area. 10. Restriction of the gathering of people.

#### 3.2.1 Management network division

The administrative boundary of the community (planning committee, village) is used as the standard to divide the basic responsibility grid, and the whole area is divided into 158 basic responsibility grids (151 in the community and village; 7 in the scenic area, and parks). And following the standard of 1,000 people, 300 households or so in the community grid set up street type, cell type, and unit type small grid a total of 722. The process of defining the boundaries of each grid involves urban roads, with the road median as the demarcation standard, and all basic responsibility grids cover every road, every building, and every facility unit.

#### 3.2.2 Grid personnel duties and work requirements

The first category is the grid manager. The grid chief is the director or secretary of the community to which the basic responsibility grid belongs, responsible for the unified scheduling of the grid administrator to carry out the work, the grid set up a unified signage, formula grid management personnel. The second category is the professional grid member. The professional grid clerk by the city, district, and street has three “ten members” (municipal, sanitation, gardening, street community, urban management law enforcement, wardens, water, electricity, communications, and traffic police). The third category is the grid responsible personnel. Grid responsibility personnel is mainly for the basic responsibility network. Major urban management problems that cannot be disposed of promptly within the basic responsibility grid should be established as a transfer mechanism and transferred to the relevant units on time, and the relevant units should organize emergency mobile forces for timely disposal to ensure good order within the basic grid.

## 4 Triple role play of “social safety valve” for grid management

The complete risk management process starts with risk identification, based on risk evaluation, risk warning, crisis response, and continuous improvement at all stages of risk management through continuous monitoring and review. Risk identification is the starting point for effective prevention and control of community risks. Due to the complex composition of community members and trivial matters, we often reacted passively in the past due to the inability to grasp the source of risks. In the “First National Governance Summit Forum and Top 100 Outstanding Achievements in Governance Innovation,” the “Yuelu Model,” with its grid management, social services, information support, and front-line law protection, has been bravely explored in Changsha’s Yuelu District. The “four-in-one” social governance model was awarded the title of “Top 10 Social Management Innovation” in China. The role of Grid Management is shown in Table 2 below:

**Table 2:** Triple role play of “Social Safety Valve” for grid management.

Function	Benefits	Concrete Measures
Community Stress Reduction Function	It uses flat management tools, which are mainly reflected in the flattening of the management model and management process, improving the agility, precision, and efficiency of management. It releases the original management pressure and resolves internal conflicts.	1. “A + B + C + D” Model of Grid Management 2. First-line Method Guarantee
Community Alarm Function Community Integration Function	It proactively identifies problems and solves them, completely changing the old management model of reacting to problems. It is a scientific closed management mechanism, not only with a set of standardized and unified management standards and management processes but also the information collection, assessment and evaluation, verification and closure, disposal feedback, command and dispatch, and case file establishment of the six steps to form a closed loop, thereby greatly enhancing the ability and level of management.	1. Management Warning 2. Technical Early Warning 3. Manual Warning 4. Regular Alerts 1. Integration of Organizations 2. Integration of Information 3. Integration of Personnel

### 4.1 Community stress reduction function

Social stress reduction, that is, to reduce or alleviate the hostility between groups in social conflicts. The stress reduction function, which provides a mechanism for draining hostile and aggressive emotions and preserving the social system by organizing possible conflicts in other parties or mitigating their destructive effects, thus releasing the closed tensions, is the most fundamental function of the safety valve system and the basis on which other functions can be performed. The “Yuelu model” grid management has achieved “Two Models”:

#### 4.1.1 “A + B + C + D” model of grid management

Urban management grid management issues are divided into four categories according to the level of disposal matters: A category for the basic responsibility grid responsible for disposal matters; B category for the street with the basic responsibility grid responsible for disposal matters; C category for the city and district management departments linkage disposal matters; D category for the municipal departments’ disposal matters.

A category problems are self-disposed on-site, B category problems are reported to the street grid platform for assignment to relevant departments, and C category problems are reported to the district city management platform for task assignment. Finally, D category problems are reported to the city management platform for disposal by the respective departments. This framework ensures effective and efficient management of urban issues through the grid system.

#### 4.1.2 First-line method guarantee

The aforementioned measures effectively mitigate community risks through various strategies.^2^ Firstly, by adhering to the principles of “fixed point, fixed person, and fixed time,” leadership cadres establish direct connections with grassroots communities, promptly addressing their needs and resolving potential risks. Secondly, the implementation of a networked community management system fosters seamless collaboration among district-level leadership, functional departments, and community section cadres, facilitating efficient resource allocation and risk management. Additionally, information regarding community management divisions and cadre responsibilities is transparently publicized through channels such as publicity windows, electronic display screens, and signs. This ensures accountability and enables quantifiable evaluations of cadre performance, focusing on their proactiveness, problem-solving abilities, and community satisfaction. These comprehensive approaches enhance the coordination, transparency, and effectiveness of community risk management, ultimately reducing the potential impact of risks within the community.

Yuelu District Administration has solved the problems of the public promptly. As of November 17, 2020, the council received and processed a total of 21,824 work orders for complaints and reports, which have been completed by 20,750, with a completion rate of about 95.08℅ and a satisfaction rate of more than 98%. Importantly, there have been no collective letter and petition incidents and public opinion incidents have occurred.

### 4.2 Community alarm function

The social alarm function means that the social manager or the ruling class can observe and understand the people’s situation and estimate the level of social group conflict through the “safety valve.” The “Yuelu model” grid management has achieved “Four Major Warnings”:

First, management warning. In the “Yuelu model” of urban management grid management issues are divided into four categories according to the level of disposal matters, that is, according to the number of social contradictions, work tasks, and other factors, all grids are divided: A for the basic responsibility of the grid responsible for disposal matters; B for the street with the basic responsibility of the grid responsible for disposal matters; C for City and district management departments linkage disposal matters; D class for the municipal department’s disposal matters. By configuring different levels of risk disposal powers, the disposal of unexpected events at different levels can be achieved.

Second, technical early warning. The “Yuelu Model” also applies technical means to monitor risk parameters. For example, Xianjiahu Street has established a “sky network” and “ground network” monitoring system. Xianjiahu street “sky network” into the street comprehensive governance command center platform and community sub-control platform, “ground network” is installed in the jurisdiction of the intelligent face card camera and face recognition integrated control platform, to achieve full monitoring of the main roads, community entrances, and exits, etc. Coverage, “people-events-things” dynamic management, the formation of “sky-ground” remote echo of the work pattern.

Third, manual warning. The basic process of dealing with grid matters in Yuelu District is that each grid responsible person inspects the responsible grid during working hours, finds out all kinds of urban problems in the grid the first time, and takes self-disposal, telephone report, WeChat report, etc. to dispose of the problems in time. The grid platform categorizes and manages tasks into four levels: A, B, C, and D, enabling a shift in community management from reactive problem-solving to proactive issue detection, and from post-enforcement to front-end service management.

Fourth, regular alerts. On the 28th of each month, leadingin direct communication with the masses during their stationing. On the stationing day, the convener of each village and community grid are responsible for holding a face-to-face meeting to discuss the collected issues and propose feasible solutions. The stationing day is warm for party members to contact the masses and cadres to face them directly. Based on the risk analysis, the network management will issue regular alerts on the type, cause, manner, location, extent, and frequency of potential risks, which will help alert people to take effective prevention and control measures.

### 4.3 Community integration function

The community integration function is to promote community integration, that is, conflict drives social groups to divide, and divided groups come together and solidify into groups. For social groups, releasing tensions can remove the divisive elements of hostile relationships and reestablish unity, thus serving the function of intra-group cohesion and integration. The “Yuelu model” grid management has achieved “Three Integrations”:

#### 4.3.1 Integration of organizations: “1 + N” socialized services

**Figure 3:**
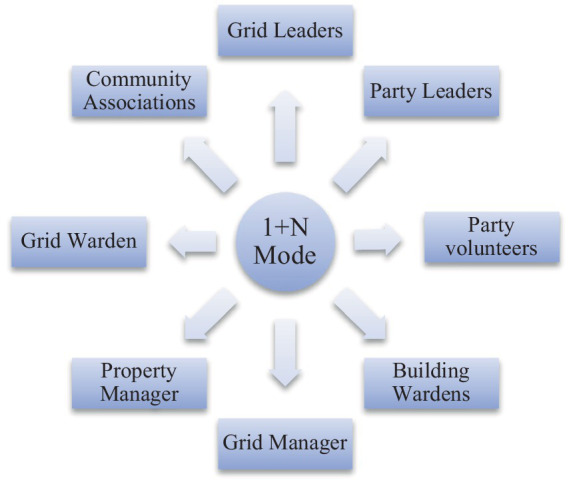
The “1 + N” working model.

Socialized service is the integration of resources to provide diversified services. Grid is the carrier; service is the purpose. Social governance emphasizes the participation of multiple subjects, especially to stimulate the vitality of society. The “Yuelu Model” proposes grid management, and the “1 + N” working model (Figure 3) puts matters closely related to the people in the grid area to specific people, effectively identifying risks and helping to extinguish the “fuse” of “burning the “fuse” of the “substance.” In the “1 + N” model, the main role of the people in the community risk management is highlighted, the grid leader and the grid supervisor, grid manager, grid public information officer, grid security officer, respectively, by the street (town) cadres, community (village) members of the two committees, the street (town) mobile population and rental housing coordinators and household police The “four members,” together with “N” represents the social forces of building leaders, party volunteers, information teams, residents’ associations, constituting the “eyes” to find sources of danger, feedback problems “and “tentacles” (12). Gird management extends grassroots work to all parts of society, from “outside the walls” of the neighborhood to “inside the walls” of the residential area in the management area, and from “8 h” to “24 h” in the management time. The management time is extended from “8 h” to “24 h,” and the social control covers all aspects and elements of “people, things, objects, and organizations,” weaving a meticulous “risk network”, that is, as long as there are hints of harm to community safety, immediately trigger the response of the grid personnel, to maximize the elimination of management blind spots and management dead ends, prevention and control of risks and hidden dangers.

#### 4.3.2 Integration of information: information technology support

**Figure 4:**
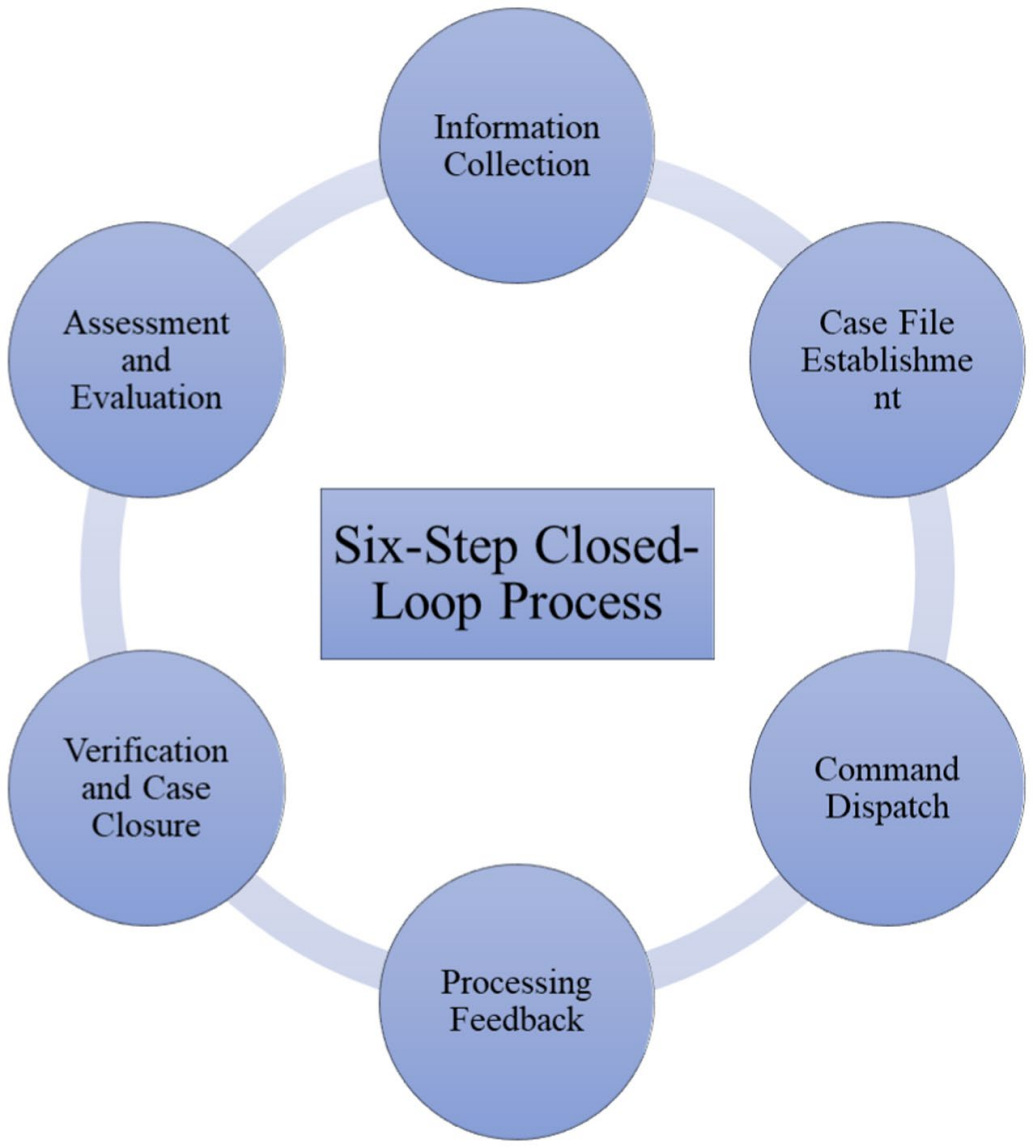
The “Six-Step Closed-Loop Process” model.

Build a platform to implement fine management. To maximize the effectiveness of social governance, Yuelu District makes full use of modern information network technology to develop a high-level comprehensive, integrated, and shared network information system to implement full-time monitoring of the grid and connect the network and transfer information to the street (township) and each community (village) to provide a network service platform for social management services. At the same time, it is designed with five modules of general security, urban management, party building and group, economic management, and social affairs, involving 34 major categories, 125 sub-categories, 468 sub-categories, and 693 sub-services. By implementing a six-step closed-loop process of information collection, case establishment, command dispatch, feedback handling, case verification, and assessment, a classification and time-limited processing system is established, ensuring the orderly operation of the network system and enabling precise and efficient management services. As shown in Figure 4 below:

#### 4.3.3 Integration of personnel: risk governance subject

The grid management model can effectively extend the chain of social governance and mobilize more people to join the community governance team consciously. The X service model of “one grid, one party group, one autonomous organization, and one volunteer team or association” not only reduces the cost of social management by the party committee and government but also helps the public to improve their ability to educate themselves, manage themselves and solve problems on their own. Some communities have made efforts from “hard power,” not only introducing, and cultivating several high-quality property management enterprises, but also cooperating with small and micro enterprises closely supporting community life, to provide better services for community residents; some communities have improved from “soft power.” The development and growth of arts and culture teams, mainly art lovers, have greatly enriched the cultural life of the community. Gird management has changed the previous state of life, where people knew what they were doing, and now they have someone to take care of everything, which has relieved grassroots conflicts and smoothly expressed their interests. The relationship between the cadres and the community has become closer, and the satisfaction and happiness index of the residents has been greatly improved.

## 5 Results

**Figure 5:**
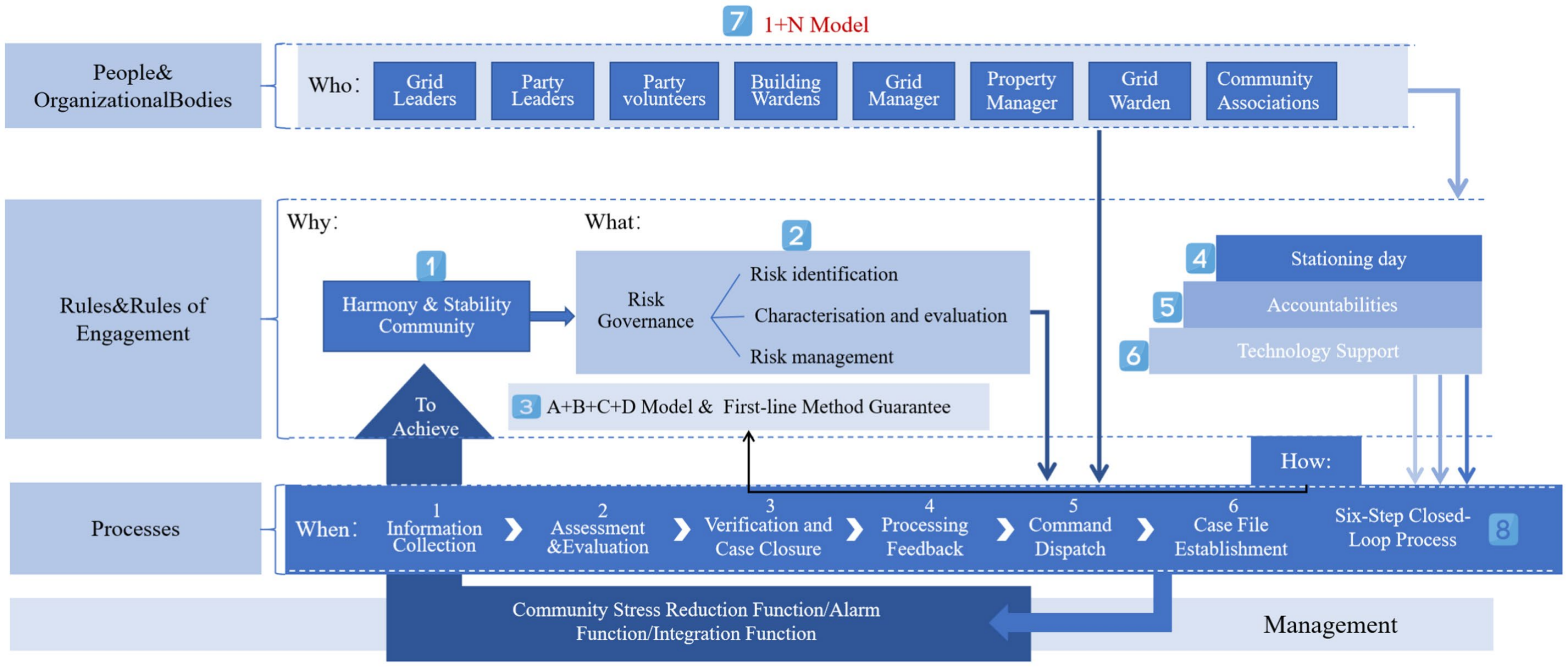
The risk governance logic and pathways of the “Yuelu Model.”

The “Yuelu Model” systematically deploys and coordinates arrangements at three levels, optimizes, integrates multiple resources, and achieves rapid linkage (Figure 5). By fully mobilizing units and functional departments to participate in and support social management work, information from public security, family planning, civil affairs, social security, and other departments are incorporated into a comprehensive resource base, facilitating effective information sharing. This model breaks down the division of neighborhoods and establishes a comprehensive set of basic information databases that integrate population, unit information, and housing information, covering science, education, culture, and health, etc.; secondly, integrating organizational resources, generally establishing multi-disciplinary management service teams composed of government organizations, social organizations, and private enthusiasts, and enhancing the overall service capacity of the service teams; thirdly, integrating social resources, encouraging the participation of civil organizations and the public and give full play to the power of social capital. For example, the organization of retired older person comrades in the community set up a community volunteer patrol team to investigate the hidden dangers of law and order; serve as “Internet volunteer supervisors,” and regular conducting volunteer supervision in local Internet cafes.

In short, the social safety valve ensures the reconstruction of the social system by using conflict as a method and a means to mitigate tensions and resolve conflicts, thus maintaining the stability of the social structure. In this process, the decompression function of the community is a prerequisite and basis for the implementation of other functions, the integration function of the community is implemented by controlling the rational emergence of conflicts, re-regulating the relationship between groups in the community structure, making the initial observation and clarification of the situation of the inhabitants, and preventing the degree of conflict emergence, i.e., the social warning function. These three functions do not operate in isolation from each other. But are closely linked on the logical basis of the conflict-positive function and operate progressively as community members interact.

## 6 Discussion

At present, the comprehensive construction of a moderately prosperous society has entered a decisive stage. Community safety is a basic requirement for the happiness and well-being of residents, a prerequisite for the development of grassroots society, and an important cornerstone for the stability of the state. Enhancing the awareness of worry and risk, and being prepared for danger in times of peace and preparedness is a major principle that must always be adhered to in the process of social governance. For more accurate and scientific community grid management and effective risk prevention and control, we also need to enhance risk awareness, take advantage of new technologies, and further optimize risk prevention and control mechanisms.

First, based on big data as a platform, a shared information base is established among various departments. Through the mastery of the risk factors identified and analyzed, manual dynamic identification and information sharing of possible risks are carried out in the process of risk management in due course. Led by the Comprehensive Governance Committee, the public security organs play the role of the main force, the relevant departments work closely together, relying closely on grassroots organizations, and strive to form a multi-participation and shared work situation.

Second, through multi-departmental linkage to establish a dynamic government intranet of public information, the application of technical means, through risk analysis, set up automatic detection technology system to detect risk parameters, that is, the “security threshold “, and issue risk warnings when the set security threshold is reached, to guide the relevant departments to carry out risk prevention and control.

Third, establish a regular (or periodic) warning mechanism. Based on risk analysis, regular (monthly, quarterly, or yearly) early warnings on possible risk types, causes, ways of occurrence, location, degree, frequency, etc., are issued to draw “risk maps” to raise awareness and guide the relevant departments to take effective measures to prevent and control.

Fourth, to provide regular training for grid supervisors, grid administrators, grid public information officers, and grid security officers to establish a high-quality grid management talent team. The implementation of comprehensive risk prevention and control mechanism is inseparable from the quality of the talented team of each department, through regular job training mechanisms for assessment, improving the alertness and identification ability quality of the grid management personnel, to ensure the lean and excellent risk prevention and control team, to ensure the successful implementation of the risk prevention and control mechanism in the process of social governance.

From the analysis above, it can be seen that the grid based management mechanism has shown strong adaptability in responding to community risks. While seeing its positive effects, we should also note that the grid management model has risks and defects. The current grid management is not proposed to be established under the framework of adequate theoretical research and then put into practice, and there are inherent risks and defects in the management model itself. The grid management of urban communities is a three-level organizational structure composed of streets, communities, and grids, which is equivalent to adding a governance level beyond streets and communities and extending the chain of social governance. Under this new organizational structure, the specific task of realizing community governance is in the three-level grid, i.e., residential neighborhoods, buildings, and units in the community (25). Although there is no substantial institution in the lowest level of the three-tier grid, the decentralization of administrative resources and grid community party building have increased the difficulty of information transmission, demand response, and functional coordination in the governance process. Therefore, because of the increase in the governance level, the corresponding management institutions should be increased. For example, some cities have set up management agencies for community grid management of the resident grid members, grid members, etc. To carry out unified management. This will inevitably cause a series of problems such as the increase of administrative layers and the expansion of institutions. The increase in personnel due to the increase in the number of layers and the expansion of agencies further aggravates the financial burden, thus forming a vicious circle (26).

## 7 Conclusion

Social stability is the prerequisite for national strength, and grassroots governance is the cornerstone of national governance. Urban community grid management systems often become self-reinforcing, hindering community autonomy and self-governance. The administrative-led nature conflicts with residents’ aspirations for autonomy and rights (18). This weakens community participation, social organizations, public spirit, innovation, and vitality (27). Without timely adjustments, it impacts effectiveness and hinders China’s grassroots social governance. Analyzing risk prevention, identifying sources, and enhancing measures are crucial for a cooperative governance model. This builds a harmonious society in the long term.

With over 10 years of grid management practice, the “Yuelu Model” has emerged, incorporating grid management, social services, information support, and front-line legal safeguards into a unified framework, promoting a comprehensive and integrated approach to grid management services. Undoubtedly, the success of the “Yuelu Model” grid management clearly demonstrates the significant role played by grid management in community risk prevention and control. Grid management is an innovative approach to grassroots social governance. To strengthen the grid management for community prevention and control, it is necessary to leverage information technology as a key driver and pursue refined management as the objective. By fully integrating grassroots resources, we can achieve precise risk prevention and control in the community.

## Data availability statement

The original contributions presented in the study are included in the article/supplementary material, further inquiries can be directed to the corresponding author.

## Author contributions

SG: Conceptualization, Methodology, Validation, Writing – review & editing. WL: Data curation, Investigation, Software, Writing – original draft, Writing – review & editing.

## Footnotes

^1^Social governance “Yuelu Model” won the national top ten, Changsha Evening News. Retrieved 22/07/2021 from https://www.icswb.com/newspaper_article-detail-22828.html.

^2^Grassroots Practice and Reflections on Gird Management Services in Social Governance Innovation - Taking the Urban Area of Changsha City as an Example, Journal of the Party School of the Sichuan Provincial Committee of the Communist Party of China, 2014 Zhang Kelan.
